# How ignoring the harm of killing undermines the ethical legitimacy of animal research: a case study from Switzerland

**DOI:** 10.1007/s11019-026-10382-y

**Published:** 2026-07-27

**Authors:** Christian Rodriguez, Charlotte E. Blattner, Angela K. Martin

**Affiliations:** 1 Institute for Biomedical Ethics, University of Basel, Basel, Switzerland; 2 Faculté de droit, des sciences criminelles et d’administration publique (FDCA), Université de Lausanne, Lausanne, Switzerland; 3 Environmental Sciences and Humanities Institute, University of Fribourg, Fribourg, Switzerland

**Keywords:** Animal research ethics, Harm-benefit analysis, Euthanasia, Killing animals, Animal welfare, Animal law

## Abstract

The most established approach to legitimizing animal research worldwide is through Harm-Benefit Analyses (HBAs), which require that the expected benefits of research be compared to and outweigh the harms done to animals. For this process to be ethically sound, all relevant harms must be considered. However, HBAs leave one fundamental harm insufficiently considered: killing, i.e., the deliberate termination of animals’ lives as part of the research project. This article argues that the law’s ignorance of the harm of killing leads to its trivialization in practice and, consequently, undermines the ethical legitimacy of deadly animal research projects. While our argument applies broadly, we use Switzerland as an illustrative case example. We first disentangle, from an ethical standpoint, the harms that killings inflict on animals in research. Second, we show that most harms of killing, both to the inherent worth and wellbeing of animals, are not accounted for in law, allowing killings to be trivialized. Third, we propose that killing should always (a) be subject to ethical and legal scrutiny by animal research commissions, (b) have to pass the necessity test (i.e., killing should be permitted if, and only if, it is necessary to achieve the purpose of the experiment), and (c) be included in HBAs. Fourth, we consider common objections to our arguments, and, in concluding, show why recognizing the harm of killing is essential to upholding the ethical legitimacy of animal research.

## Introduction

Most laws governing animal research seek to justify the use of sentient non-human animals (henceforth: animals) in research by a process known as “Harm-Benefit Analysis” (HBA) (ECAE 2022; EU 2010; NHMRC 2013). HBAs require sufficient knowledge of the expected benefits of a planned research project and the likely harms to affected animals, to balance the two. HBAs are “successful”, for research, if they demonstrate that the benefits outweigh the harms (Grimm et al. 2018).

For this process to be sound (i.e., methodologically, ethically, and legally valid and justified), it is necessary to consider all harms incurred by the animals. Failing to recognize some harms to the animals would undermine the ethical justification of an experiment^1^. Many states detail HBA requirements in either laws or guidelines, including on how to evaluate harms likely to be inflicted on animals (ECAE 2022; GIRCOR 2021; NSW 2023). Yet, there is one fundamental harm that largely remains unaddressed, and, accordingly, unevaluated, by HBAs: killing, i.e., the deliberate termination of animals’ lives as part of the research project.

This neglected aspect of HBAs is ethically significant. The overwhelming majority of animal ethicists consider premature death, if not in the interest of the animal in question (e.g., not due to unbearable suffering), a significant harm. To completely ignore the harmful killing of animals in research amounts to a neglect of the basic requirements of an HBA, and hence, of an ethically justifiable analysis. In practice, this leads to several problems. Professionals (e.g., researchers, commission members, judges) face practical difficulties in assessing the harms of experiments, and practitioners are asked to deal with the burden of causing animal death (e.g., psychological burdens). More fundamentally, the inadequate consideration of the harm of killing leads to skewed HBAs, and a trivialization of killing in practice, which in turn, undermines the ethical legitimacy of deadly animal research projects.

To illustrate this problem, we critically examine the laws of one of the world’s leading countries in protecting animals, namely, Switzerland (World Animal Protection 2025). We specifically zoom in on the Swiss legislation because, contrary to many other countries, it is dedicated to protecting the dignity of animals, in addition to their wellbeing.^2^^,^^3^ This allows the legislation to account for both sentientist harms to animals (that is, harms directly experienced by them) and non-sentientist harms (harms not directly experienced by animals) (ECAE 2022). As we will show, this particular feature of Swiss laws (also existent in other jurisdictions, such as Liechtenstein, Luxemburg and South Korea) provides a clear legal basis for considering the harm of killing in all its dimensions (Liechtensteinische Landesverwaltung 2010; Grand Duchy of Luxembourg 2018; Republic of Korea 2023). This article will demonstrate that even the relatively strict Swiss laws fail to adequately consider this harm.

Despite our focus on Switzerland, our analysis is relevant for any country that employs the HBA to evaluate animal research projects. Whether or not a given legal framework distinguishes between sentientist and non-sentientist harms, failure to account for the harm of killing undermines the process of HBAs, and consequently the legitimacy of deadly animal research projects. In this sense, our recommendations for the Swiss context can help inform legislation in other jurisdictions that rely on HBA-based evaluations of animal research.

For our analysis, we will consider the various types of killing in research and their associated harms. Drawing on Persson and colleagues’ categories (Persson et al. 2025), we examine five types of killing that occur at different stages of research, from animals killed during experiments, to those killed after the study is completed or as part of breeding practices. As we will show, each category gives rise to different combinations of harms.

We beginin by defining when, why, and to what extent killing is a harm in animal research from a normative perspective. We then show how current Swiss animal research laws and guidelines fail to consider most of the harms of killing. Next, we propose changes to Swiss law to better address the harm of killing. We take two cases (i.e., genetically unfit animals and so-called “leftover animals”) as examples of why such changes are needed: economically driven killings, though unnecessary and ethically questionable, remain common and legally allowed in animal research. On this basis, we recommend that (i) killing should always be subject to ethical and legal scrutiny by commissions, (ii) killing should have to pass the necessity test (i.e., killing should be permitted if, and only if, it is necessary to achieve the purpose of the experiment), and (iii) that (all) killing(s) should be considered in HBAs. Finally, we consider the most potent objections to our arguments, before concluding our analysis.

## When, why, and to what extent is killing a harm to animals in research?

The ethical implications of killing animals constitute a major concern in animal ethics (Višak and Garner 2015; Meijboom and Stassen 2016). There are various positions defending the view that premature death harms animals, even if death occurs without any pain and suffering. According to a desire-satisfaction approach, death harms animals because it deprives them of satisfying future desires (such as spending time with others, investigating their surroundings, eating, and the like) (Singer 2011; Martin 2022). According to the deprivation view, death harms animals because it is the ultimate form of harm: it deprives them of their existence and forecloses all opportunities for future experiences (Regan 2004; Sapontzis and Steve 1987; DeGrazia 1993; Rollin 2006). According to the time-relative interests account, not only the amount of time lost due to death matters but also one’s psychological continuity over time: the more I am connected with my future self and have made plans for the future that are frustrated by death, the worse is death to me (McMahan 2015). This view has important implications for animal research, as it implies that the wrongness of killing varies among species in accordance with their cognitive capacities.

Whilst it is outside the scope of this paper to further add to this debate about the harm of killing animals, it is important to note that, based on the above-presented accounts, an overwhelming majority of animal ethicists consider killing, if it is not in the interest of the animal in question (e.g., due to unbearable suffering),^4^ a significant harm. For the sake of the arguments presented here, we thus start from the assumption that killing *is* a significant harm to animals.

Likewise, it is beyond the scope of our analysis to challenge the narrow ethical focus underlying current animal research laws and the HBA. Although not explicitly utilitarian, the HBA is primarily concerned with the quantification of harms and benefits (Müller and Katz 2026). Moreover, as discussed below, the notion of *harm* in this context is largely grounded in a sentience-based approach (Blattner 2019a). Sentience can be defined as the capacity to have consciously experienced states with positive or negative valence (i.e., experiences that feel good or bad to the subject), such as pleasure and pain (Birch 2024). There are good reasons to argue that other ethical frameworks instead of the HBA would be more appropriate for governing the use of animals in research. For example, some frameworks articulate principles for research grounded in respect, justice, and animals’ participation (Van Patter and Blattner 2020), while others extend human research principles to animal research (Martin 2022). Within such approaches, deontological constraints play a significant role and can render the killing of animals unjustifiable, thereby requiring a fundamental reconsideration of animal research governance.^5^ The present paper does not engage with these broader critiques of current animal research practices. Instead, it operates within existing legal and regulatory structures and seeks to demonstrate that, even on their own terms, they require a more rigorous and explicit incorporation of the harm of death.

Since Switzerland serves as our illustrative case example, we will base our argumentation on the moral status assigned to animals within this specific context – that is, a lower moral status than humans (Bolliger 2016) – which is the case also for most other jurisdictions. In this section, we argue that the moral status given to animals in Swiss law logically implies that all killings in animal research *should* be considered a harm, and some of them to a greater extent. Note that our argumentation *ceteris paribus* also applies to other legislations with similar assumptions (such as animals’ intrinsic value and/or dignity).^6^ We will specifically focus on the harms to the *animals*, but it should be mentioned that professionals working in the field of animal research may also be harmed by the trivialization of killing that we discuss in this article (Grimm 2023).

Since 1992, introduced by popular vote, the Swiss Federal Constitution (Cst) (Swiss Confederation 1999) recognizes nonhuman animals as worthy of protection and (just like any other living creature) as having an *inherent worth*.^7^^,^^8^ Consequently, the Swiss Animal Welfare Act (AniWA) (Swiss Confederation 2005) is dedicated to protecting the dignity of animals, in addition to their wellbeing. Per the AniWa, this protection is limited to *sentient* animals.^9^

We recognize three ethical implications of the inherent worth of sentient animals in this context. First, their sentience gives them the status of, at least, *moral patients*. In other words, sentient animals matter for their own sake, can be harmed, and deserve moral concern (Gruen 2003). Second, animals’ inherent worth must be respected. All sentient animals share a similar worth that is not dependent on any other specific capacities (e.g., evolutionary proximity to humans, intelligence, aesthetics, or relational ties with humans) (Francione 1995). Third, it matters to sentient animals if their wellbeing is affected. This means that sentient animals have interests related to their wellbeing (e.g., not experiencing pain) and these interests may differ depending on their other capacities (e.g., capacity for social relationships). This is in line with dominant welfarist approaches in animal ethics (Višak and Garner 2015; Singer 2009).

Let us now make some considerations about death. Death, for animals, equates to their life ending. Thus, death implicates the end of their existence, sentience and of their wellbeing (i.e., an end to both positive and negative subjective experiences). Three distinctions must be made in this context. First, some deaths involve sentientist harms (i.e., harms subjectively experienced by the animal during death) while others (at least in theory) do not. Indeed, the way in which death happens may or may not involve negative subjective experiences (e.g., pain, anxiety and distress). If an animal is killed with a suboptimal method and does not die immediately, the animal may suffer significantly during the last minutes of their life. An example is death by suffocation or drowning (EFSA 2015, 2020a, 2020b). Other forms of death, however, do not entail any sentientist harm – the animal does not experience any pain, distress, anxiety, or suffering. This is for example the case if the animal is dying while unconscious. Second, some deaths qualify as killings whilst others do not. Killing can be defined as the intentional infliction of death, whilst dying often is the unintended consequence of other actions. In the context of animal research, animals can die (e.g., in facilities due to unforeseeable health issues) or be killed (e.g., for tissue collection, upon finalization of a research project). Third, some killings are cases of euthanasia and others are not. Euthanasia of animals can be defined as “the (painless) killing of an individual by a veterinarian, if ending its life is presumably in the individual’s interest” (Persson et al. 2020). Conversely, if death is not in an animals’ ultimate interests (e.g., not due to unbearable suffering that cannot be alleviated), then the animal is killed. In the remainder of this paper, we will focus on *killing* and will only refer to unforeseeable death or euthanasia for specific examples.

With regards to the animal’s *inherent worth*, some killings appear to be a greater harm caused to the animals, because their cause is trivial. For example, killing animals for convenience (e.g., economic and logistic reasons) fails to respect their inherent worth to a greater extent than killing animals for biomedical reasons (e.g., tissue analysis necessary for medical research).

In relation to animals’ *wellbeing*, it matters whether one considers it important that an animal can self-consciously envision their future. On the one hand, if one thinks that having future-related anticipations, desires, and hopes does *not* matter morally, then the fact that, for example, mice do not have a capacity to envision and desire positive experience that will happen to them in a mid- or long-term future does not minimize the fact that it is in their *interest* for them to happen. Therefore, depriving mice of those future positive experiences would constitute a harm to them in this view, even though the mice are unaware of what they lose out on. According to this view, the extent of harm depends on the extent of deprivation, meaning that both the quantity (e.g., life expectancy) and quality (e.g., animal’s capacities and potential flourishing) of lost experiences are relevant. On the other hand, if one assumes that the capacity to self-consciously envision one’s future is a necessary condition to be harmed by death, the picture presents itself differently. According to these desire-based accounts, mice cannot have an interest in the positive experiences that will happen to them in the future because they lack the psychological connectedness to these experiences. Therefore, depriving mice of those future positive experiences would not constitute a harm to them in this view. This view has important implications for species with higher capacities for self-consciousness, like primates and corvids (Selter 2020). However, one could respond to such a view by suggesting that animals lacking future-oriented self-consciousness may experience the present more intensely, which would amplify rather than diminish the welfare significance of what is lost at death (Schukraft et al. 2024). We will consider these different positions as plausible in the remaining parts of this article.^10^

Based on these considerations, four types of harms can be distinguished as part of killing in animal research. Importantly, one does not need to agree with all four harms for our argument and conclusions to remain valid.

### Harm 1 (H1)

All killings involve a *general harm* to the *inherent worth* of the animal (i.e., a fundamental deprivation). This harm is equal for all sentient animals.

### Harm 2 (H2)

Some killings involve *additional harm* to the *inherent worth* of the animal (i.e., greater failure to respect their inherent worth).

H2 is for example the case if animals are killed for trivial reasons, i.e., reasons that are insufficient from an ethical perspective. Examples are the killing of animals for logistic and economic reasons (e.g., insufficient financial resources or space, failure to look into rehoming options, and the like).

### Harm 3 (H3)

Some killings involve harm as a *wellbeing deprivation* (i.e., deprivation of future wellbeing, and experiences). The extent of this harm may differ according to the animals’ capacities.

### Harm 4 (H4)

Some killings involve *sentientist harms* to animals (i.e., deaths involving suffering).

In practice, different categories of killing animals in research will cause some, or potentially even all, of these harms. Adapted from Persson et al.’s main categories of killing animals in research (Persson et al. 2025), Table 1 below gives an overview of the categories of killings and their potential harms.

**Table 1:** Categories of killings in research and potential harms (adapted from Persson et al. 2025)

Category	Description	Potential harms
Killed during experimentation	Animals killed during research for scientific reasons (e.g., tissue collection)	H1: Fundamental deprivation
		H3: Wellbeing deprivation
		H4: Sentientist harm
Killed in advance for use	Animals killed before research to use their bodies (e.g., organs, tissues). This includes in vitro research based on animal body parts.	H1: Fundamental deprivation
		H3: Major wellbeing deprivation (killed early)
		H4: Sentientist harm
Killed as surplus or no-use	Animals killed before entering the research stage because they either do not have the desired characteristics (e.g., failed genetic modification), or were “stock supply”.	H1: Fundamental deprivation
		H2: Major harm to inherent worth (killed for trivial reasons)
		H3: Major wellbeing deprivation(killed early)
		H4: Sentientist harm
Killed after being used for breeding	Animals used for breeding only (i.e., never supposed to enter the research stage) and killed once not needed or not productive anymore (e.g., for economic or logistic reasons).	H1: Fundamental deprivation
		H2: Major harm to inherent worth (killed for trivial reasons)
		H3: Wellbeing deprivation
		H4: Sentientist harm
Killed as leftovers after study	Animals who have a sufficient quality of life when research is over, but are still killed (e.g., for economic or logistic reasons).	H1: Fundamental deprivation
		H2: Major harm to inherent worth (killed for trivial reasons)
		H3: Wellbeing deprivation
		H4: Sentientist harm

This analysis shows that different categories of killing can cause distinct harms to animals, affecting both their inherent worth and their wellbeing. This has important implications for the evaluation of animal research projects through HBA. Indeed, most laws governing animal research require that all harms to animals be considered and weighed against the anticipated benefits of the research (ECAE 2022; EU 2010; NHMRC 2013). To meet this standard, a nuanced assessment of the harms of different cases of killing is necessary, including by the law. Therefore, animal research laws and guidelines, particularly those concerning HBA, should incorporate a nuanced understanding of the various harms of killing. This approach aligns with the view that ethical considerations should guide the law in determining which aspects are weighed in HBAs.

## Trivialization of killing: gaps in Swiss laws and guidelines

So far, we have established that killing animals causes them in many cases a serious harm that is ethically relevant. In what follows, we turn to the law, specifically animal research law, which defines the basic parameters of HBAs, to investigate how it deals with the harm of killing in animal research. Our analysis zooms in on Swiss law, considered one of the most advanced worldwide (World Animal Protection 2025). The aim of this section is to show that most harms of killing defined above fail to be mentioned and considered in Swiss laws and guidelines.^11^ As we will show, only H2—killings that represent a significant failure to respect the animal’s inherent worth—is covered by Swiss guidelines, whereas other harms (i.e., H4) are insufficiently addressed, and some (i.e., H1 and H3) not addressed at all. The fact that one of the most progressive animal research laws in the world fails to address the fundamental harms inflicted on animals in research is indicative of the likely failure of other jurisdictions to do the same, and exemplary of the larger failure of animal law to effectively protect animals. Our analyses are likely easily applicable to other legal contexts. Overall, this failure to recognize and differentiate between the harms of killing undermines the ethical justification and legitimacy of animal research more widely and results in its trivialization in practice, as we will argue.

In addition to its constitutional foundations, Swiss law on animal research is mainly composed of the AniWA, the Animal Protection Ordinance (AniPO) (Swiss Confederation 2008), and the Animal Experimentation Ordinance (AEO) (Swiss Confederation 2010). As previously stated, these laws have the purpose to protect the dignity and wellbeing of animals (Bolliger et al. 2019). To achieve this dual purpose of protecting the dignity and wellbeing of animals, the AniWA defines the terms “dignity” and “wellbeing” as follows:

AniWA, Art. 3, *“Definitions”*:^12^

- Dignity means the inherent worth of the animal that must be respected when dealing with it. If any strain imposed on the animal cannot be justified by overriding interests, this constitutes a disregard for the animal’s dignity. Strain is deemed to be present in particular if pain, suffering or harm is inflicted on the animal, if it is exposed to anxiety or humiliation, if there is major interference with its appearance or its abilities or if it is excessively instrumentalized;
- Wellbeing: the wellbeing of animals exists if:
  1. husbandry and feeding are such that their bodily functions and their behaviour are not disturbed and excessive demands are not made on their capacity to adapt,
  2. species-specific behaviour within the limits of their biological capacity to adapt is guaranteed,
  3. they are clinically healthy,
  4. pain, suffering, harm and anxiety are avoided;

As we can see, dignity is mainly defined in terms of harms that should be avoided (i.e., pain, suffering, harm, anxiety, humiliation, major interference with appearance or abilities, and excessive instrumentalization). While some of these harms (i.e., humiliation, major interference with appearance or abilities, and excessive instrumentalization) are non-sentientist (i.e., not necessarily subjectively experienced by the animal), some (i.e., pain, suffering, harm, and anxiety) are sentientist (i.e., subjectively experienced by the animal) and are also included in the definition of wellbeing. In this sense, wellbeing is one component of dignity (Bolliger et al. 2019). In what follows, we will continue using this distinction between “sentientist” and “non-sentientist” harms (ECAE 2022).^13^

Killing is not mentioned as a harm in the definitions of dignity and wellbeing, neither in terms of sentientist harms nor non-sentientist harms. While death, killing, or euthanasia are mentioned elsewhere in the law (e.g., AniWA Art. 24, 26, AniPO Art. 16 para. 2, 118, 125, 135, 177-179a), none of them demand, or offer guidance for, considering the harm of killing. For example, AniPO Art. 118a para. 3 states that surplus animals must be killed if they cannot be used for other purposes. While this provides a *legal* basis for justifying their killing, it does not help assessing the harm of killing these animals. Since the harms of killing cannot be explicitly found in these definitions, let us have a closer look at the harms relating to dignity and wellbeing by considering further laws and guidelines.

### On the account of dignity

On the account of dignity, and considering the inherent worth of the animal, we argued in the previous section that animal research laws and guidelines should recognize H1—that all killings involve a harm to the inherent worth of the animal (i.e., fundamental deprivation)—and H2—that some killings involve additional harm regarding the inherent worth of the animal (i.e., greater failure to respect their inherent worth). Yet, only the latter is recognized in Swiss law.

As mentioned above, non-sentientist harms to dignity in Swiss law include humiliation, major interference with appearance or abilities, and excessive instrumentalization.^14^ Guidelines from the Swiss Academy of Medical Sciences state, by referring to the Swiss Federal Food Safety and Veterinary Office (FSVO), that the killing of female offspring when only males are needed (and vice-versa), in the context of breeding for experimentation, qualifies as an excessive instrumentalization (ECAE 2022). In Table 1 above, this type of killing corresponds to the category “Killed as surplus or no-use”. This shows that Swiss authorities recognize certain categories of killings to be harmful to animal dignity, for example in terms of excessive instrumentalization. This consideration is consistent with our claim H2—that some killings involve a greater harm to the inherent worth of the animal (e.g., killing for trivial reasons). It also aligns with recent arguments that the killing of surplus animals should be recognized as a non-sentientist harm and that current Swiss practice underestimates the harms associated with both the housing and killing of animals who are bred but not used in experiments (Eggel et al. 2026).

However, Swiss laws and guidelines do not explicitly recognize H1−that all killings involve harm to the inherent worth of the animal as a matter of fundamental deprivation. Many scholars take issue with this and advocate for a general protection of animal life, contending that any killing of animals should be justified (Kunz 2025). For instance, Gerritsen (2021) as well as Bolliger et al. (2019) have argued that Swiss law is inconsistent in emphasizing animal dignity and welfare while lacking explicit protection of animal life. This omission undermines the very concepts of dignity and welfare, as both presuppose the protection of life itself (Gerritsen 2021). In its 2025 revised guidelines on animal experimentation, the Ethics Committee for Animal Experimentation (ECAE) of the Swiss Academies of Arts and Sciences have also discussed the question of killing in Swiss law (ECAE 2025). It stated that while the life of animals is not explicitly protected under Swiss law, “the killing of healthy animals without a particular reason can be regarded as problematic from an ethical perspective” (ECAE 2025). However, apart from emphasizing the necessity to explore other fates than death for these animals (e.g., rehoming after being used, or reuse in another experiment), no discussion of the evaluation of the harm of killing is provided. Finally, Eggel and Camenzind have argued that the forced premature termination of life (i.e., killing) is an infringement on the animal’s flourishing resulting in instrumentalization (Eggel and Camenzind 2020). On this view, killing would not be considered neutral, though the question of when instrumentalization becomes “excessive” would remain open. There is a risk that killing may be treated as no different from the mere use of animals: an instance of instrumentalization that is difficult to weigh in HBAs and often goes unchallenged.

### On the account of wellbeing

With regard to wellbeing, we argued that animal research laws and guidelines should recognize H3—that some killings entail a wellbeing deprivation (i.e., for all sentient animals according to deprivation views and for at least some according to desire-based views)—and H4—that some killings involve sentientist harms (e.g., deaths involving anxiety or pain). Yet, these harms receive little to no recognition in Swiss laws and guidelines, with the partial exception of H4.

As mentioned before, sentientist harms refer to the wellbeing of the animal. They include, among other things, the avoidance of pain, suffering, and anxiety, as well as the promotion of species-specific behavior. The wellbeing of animals is also a component of their dignity. Unlike non-sentientist harms, sentientist ones tend to be measured in Swiss animal experimentation law. The AniPO states that animal experiments must be classified by a “severity degree”.^15^ Accordingly, the AEO defines the four severity degrees as “0: no harm”, “1: mild harm”, “2: moderate harm”, and “3: severe harm”.^16^, ^17^ Killing is not mentioned as a sentientist harm to the wellbeing of the animal, and it is therefore not possible to classify it in these severity degrees only by considering these laws. However, by considering FSVO guidelines about the severity degrees, it becomes possible.

In its guidelines on severity degrees, the FSVO considers as severity degree 0 (i.e., no harm) the case of “collection of body fluids, tissues, organs, or body parts under deep general anaesthesia directly followed by euthanasia^18^ in animals not previously subjected to any intervention” (FSVO 2022a). From this example, it becomes clear that killing, assuming it is performed in compliance with approved killing methods, is not considered as a harm to wellbeing under Swiss law.^19^

This presupposes that certain killing methods are painless and that only such methods are authorized, thereby partially addressing H4. But “painless death” can be considered as a myth, as it could be argued that in practice all killing methods involve at least some level of pain, suffering, or anxiety (EFSA 2015, 2020a, 2020b; Clarkson et al. 2022). Moreover, economic and logistic incentives often trump the interests of the animal when it comes to choosing a killing method. For instance, in Switzerland the use of carbon dioxide (CO2) as a killing method, despite having raised welfare concerns, is still approved for mice and rats from the age of two weeks as “an interim solution” along FSVO efforts to find another method because “no alternative method is currently available for the euthanasia of large groups of animals” (FSVO 2022b, 2023). More welfare-compliant and ethically defensible methods do exist; however, they are not employed for large groups primarily due to economic and logistical constraints (Brunt et al. 2021).

Moreover, there is no consideration of H3—killing as a wellbeing deprivation for the animals—in Swiss law. Whether we are talking about animals who have a capacity for self-consciousness, or for social relationships, or that have a considerable lifespan, and whether they die early or late in their life, is not relevant in Swiss law since this harm of killing is neither considered from a deprivation view nor from a desire-based view. Indeed, one could argue that because the law conceives animals used for research purposes as mere means to an end, it becomes a logical impossibility to fully pay regard to the animals’ needs for wellbeing.

## Taking the harm of killing seriously: suggestions for legal reforms

As shown above, most harms of killing are not considered in Swiss laws and guidelines. The aim of this section is to show how this failure to recognize all harms of killing results in its trivialization in practice and undermines animal research’ ethical legitimacy.

If the harms of killing were recognized and implemented in Swiss law, we suggest that that the following changes should happen. First, killing should always be subject to ethical and legal scrutiny by animal research commissions (ARCs). Second, killings should pass the necessity test, i.e., all harms to the animals as part of an animal experiment must be necessary to obtain the benefits of the research. Third, (all) killing(s) should be considered in HBAs. Below, we explain these suggestions in detail.

### Killing should always be subject to ethical and legal scrutiny by ARCs

As mentioned above, all animal experiments in Switzerland must be classified by a “severity degree”. AniWA states that while all animal experiments are subject to authorization by cantonal authorities^20^, only those involving harm^21^ must be ethically and legally evaluated by animal research committees ARCs (i.e., commissions such as IACUCs composed of researchers, veterinarians, statisticians, ethicists, legal experts, or other, in charge of controlling HBAs). ARCs advise the cantonal authorities through scientific, legal, and ethical evaluation of applications, and their recommendation carries significant weight in the decision-making process (Gerritsen 2021). However, severity degree 0 experiments, which are considered as “no harm”, are not evaluated by ARCs in Switzerland. These experiments are reviewed solely by cantonal authorities^22^, and mainly from a scientific and administrative, rather than ethical, perspective. While cantonal authorities may request revisions based on scientific or administrative criteria, they do not conduct HBAs, which exclusively falls into the responsibility of ARCs.^23^ This seems reasonable at first glance: why bother doing an evaluation if no harms are involved whatsoever? The problem is that the premature death is not considered as a harm.

Swiss severity degrees only consider sentientist harms and do not consider non-sentientist ones. Killing is not considered as a sentientist harm and classifies as severity degree 0. Therefore, an experiment can entail killing and still be considered severity degree 0. The “killed in advance” category discussed in Table 1 is a typical example. Killing animals in advance to use their bodies in research is sometimes considered as a “Replacement” or a “Refinement” measure, in line with the 3Rs principles in animal research (Gerritsen 2021). Since such killings happen before any sentientist harm to the wellbeing of the animal, they are categorized as severity degree 0. In principle, this is also true for killings that suppose an excessive instrumentalization of animals (i.e., killing of female offspring when only males are needed, and vice-versa).^24^ These cases have been discussed by Gerritsen (2021), as well as the resulting problem that such killings are not taken into account in HBAs, or only indirectly via the number of animals.

Gerritsen (2021) has emphasized that it remains unclear why severity degrees refer exclusively to sentientist harms and neglect non-sentientist ones, and that it is inconsistent with the FSVO requirement to consider non-sentientist harms in HBAs. Experiments involving non-sentientist harms, such as killing, could in principle be approved by cantonal authorities without the ethical and legal evaluation of an ARC. While cantonal authorities can assign ARCs additional tasks, such as consulting them on ethically challenging severity degree 0 experiments, they are under no legal obligation to do so (Gerritsen 2021). As argued by Gerritsen (2021), non-sentientist harm, such as the harms of killing, should also be categorized in degrees of severity in our view.

In conclusion, killings of animals in research can currently be approved under Swiss law without ethical or legal evaluation by ARCs. While research teams are still required to perform HBAs for their proposed research involving the killing of animals, ARCs will not evaluate these HBAs. To take the harm of killing seriously, *all experiments involving the killing of animals should be subject to ethical and legal scrutiny by ARCs*. That is, because all killings involve at least H1—the harm of fundamental deprivation. Severity degree 0 experiment which do *not* involve the killing of animals (e.g., the animals are rehomed in a sanctuary or cared for in the facility after a harmless experiment) could still be approved by authorities without evaluation by ARCs.

### Killing should pass the necessity test

In this section, we argue that because killing is a harm, it must, like any other harm in animal research, pass the necessity test. The necessity test, in accordance with the principle of “refinement” in animal research, requires that all harms to the animals as part of an animal experiment prove necessary to obtain the benefits of the research (ECAE 2022). In other words, any harm to the animals that is not strictly necessary to obtain the benefits of the study is not permissible. In this sense, any killing of animals involving harm must be necessary (e.g., necessity to collect the animal’s organs for further analysis). Therefore, killing animals for convenience (e.g., economic or logistic reasons) is not permissible.

It is important to note that, in this context, passing the necessity test is a necessary but not sufficient condition for the legal permissibility of killing. That a killing is necessary to achieve the scientific goals of an experiment does not itself justify those goals, nor does it settle whether the experiment as a whole is ethically warranted, as that is the function of the HBA. The necessity test operates within the HBA as a threshold condition: it eliminates killings that are avoidable given the research purpose.

In our classification of the cases of killing in research in Table 1 above, we presented three categories of killings that are not necessary for the conduct of experiments nor for the sake of the animal (i.e., not cases of euthanasia). These categories are “killed as surplus or no-use”, “killed after being used for breeding”, and “killed as leftovers”. These killings are performed mainly for economic reasons and therefore do not pass the necessity test. Let us consider two cases in more detail.

First, consider the case of animals presenting the wrong genotype after a genetic modification, which fall in the “killed as surplus or no-use” category. Genetically modified animals have become widely used research models, but due to the imperfections of the process of genetic modification, many are bred without the desired genotype. This significantly increases the number of no-use animals (Ormandy 2016). Statistics from the EU suggest that while the vast majority of these animals are mice, species such as dogs or primates are also impacted (EC 2024). If the wellbeing of these animals is compromised due to the genetic modification, then their killing is a case of euthanasia and is considered a suitable option by the AniPO, art. 125. Here we are interested in all other cases, in which genetically modified animals are healthy, but killed merely for economic or logistic reasons. As noted above, the Swiss guidelines acknowledge that killing healthy animals without reason is ethically problematic, and that alternatives should be sought (e.g., rehoming, reuse), but offer little discussion on how to assess the harm of killing itself (ECAE 2025). These animals are often killed very early in life, since they cannot be used in the planned experiments, cannot be rehomed (i.e., placed in a sanctuary or a private home – which would be illegal according to most regulations on genetically modified organisms, including Swiss ones), and will not be cared for indefinitely by research facility for economic and logistic reasons (i.e., lack of resources, lack of space).

Second, consider the case of leftover animals who could continue their existence at the end of the experiment because they have not been harmed in a way that makes their lives not worth living. These cases are possible when the benefits of the research do not require the killing of the animal, for example to collect organs for further analysis. Just like in the previous case, most of these animals are killed for economic or logistic reasons. Indeed, despite initiatives aiming to increase the number of animals who can be rehomed or reused after an experiment, it is still low due to the complexity of these processes (Van Loo and Janssens 2023). One argument often put forward against this, is that most research animals are kept in poor conditions (e.g., small cages), the question being whether death is not better for them. We will discuss this objection in the next section.

In the cases described above, unnecessary killings are trivialized. Such killings are not indispensable, but do not, per standard legal understanding and reasoning, conflict with AniWA art. 17 on indispensability. Therefore, economically convenient killings are the status quo in the context of animal experimentation. As a comparison, similar economically convenient killings (e.g., killing of male chicks in egg production) have recently been prohibited in Switzerland (Swiss Confederation 2025). There are obvious parallels between these cases, and the differing legal treatment is inconsistent.

In conclusion, if Swiss law is to take the harm of killing seriously, it should require killings to pass the necessity test. Any killing (except cases of euthanasia) which is not proved indispensable to obtain the benefits of the experiments, such as the killing of wrong genotype or leftover animals, should be considered impermissible. Such animals would need to be rehomed or cared for by animal facilities. The argument usually put forward against this, is that the costs of experiments would increase and predictably fewer would take place, thus severely limiting scientific progress and research benefits for society. We will discuss this objection in the next section.

### (All) killing(s) should be considered in harm-benefit analyses (HBAs)

In the Swiss context an animal experiment is only legitimate if the harms to the animals are not “disproportionate” to the anticipated gain in knowledge. This condition is stated in the AniWA^25^ and relates to the process of “weighing of interests”, also called harm-benefit analysis (HBA). The HBA is performed by weighing the indispensable harms to the animals against the expected benefits of the research. In practice, when applying for a license, a section titled “Effects on the animals” describing these harms must be filled by researchers (FSVO 2020). In that same application, the harms must then be used to present the result of the HBA, to evaluate if the experiment is ultimately legitimate or not.

This process and the consequent legitimacy of experiments can be compromised by the lack of recognition and implementation of the harms of killing. We showed that Swiss law – as the laws of most other countries – does not recognize most harms of killing that we defined in the first section. In this sense, there are no resources to which professionals such as researchers or ARC members can refer to assess the harms of killing in different cases. Indeed, they are, *e contrario*, not permitted to do so. While they might make the judgement that the early killing of a healthy primate having social relationships in a group involves different harms compared to the killing of a quite old mouse living in solitary housing, there is no official guidance demanding that they do so, or showing them how to assess such cases in the HBA. Moreover, guidance is also needed on how to assess the harm of killing surplus animals who were brought into existence and killed, but not used in the experiment, which are often early deaths that constitute a great failure to respect the animals’ inherent worth. This lack of structured evaluation stands in stark contrast to the severity degrees of sentientist harms, where harms are systematically categorized and described in detail in Swiss guidelines (FSVO 2022a), providing a clearer framework for assessing different forms of harm.

Therefore, it is unrealistic to expect that researchers performing the HBA, as well as ARC members in charge of revising it, will have consistent and exhaustive evaluations of these harms to perform HBAs. This can lead to the approval of animal experiments without having considered all the relevant harms, or simply to inconsistent evaluations across ARCs. Moreover, recent research in the European context has shown that the reporting of harms to the animals in applications need improvement, as only a small percentage of researchers are fully reporting harms in the intended section of the document (Taylor et al. 2024).

To take the harm of killing seriously in Swiss animal research, Swiss laws and guidelines should support researchers and ARCs in the evaluation of the harm of killing to the inherent worth and wellbeing of animals. Accordingly, the relevant sections of the application to perform an animal experiment should describe these harms so that the final HBA takes all harms into account. To achieve this, a collaborative effort is needed among ethicists, animal welfare specialists, and Swiss authorities.

## Potential objections

This section considers three potential objections to our argument: that legal changes are not necessary to assess the harm of killing in animal research; that death is preferable to continued existence in animal research facilities; and that our proposal undermines scientific progress. These objections are framed within current governance frameworks and reflect concerns that policymakers and other animal research stakeholders might raise in response to our proposals.^26^

### ARCs can assess the harm of killing without the need for legal or guideline revisions

It might be argued that researchers and ARCs are already capable of evaluating the harms of killing during HBAs, even without explicit legal or regulatory guidance. While it is indeed possible that professionals exercise their own judgment in such evaluations, this approach suffers from a lack of standardization and transparency.

Currently, Swiss laws and guidelines offer a detailed framework for evaluating sentientist harms (e.g., a severity degree assigned to a specific type of surgery). However, there is no equivalent guidance on how to assess the harm of killing. This leaves professionals without institutional support or criteria to make such decisions consistently. Given that some harms of killing involve complex moral dimensions, including distinctions between sentientist and non-sentientist harms, expecting consistent judgments from ARC members with diverse backgrounds places an undue burden on the commission.

Furthermore, a legal revision is necessary to address the issue of trivialization of severity degree 0 killings. As we have shown, the killing of research animals in Switzerland can be authorized without any consideration by ARCs, even when they constitute significant non-sentientist harms (e.g., excessive instrumentalization). To rectify this, legal changes are required to ensure that all experiments involving the killing of animals, regardless of their severity degree, are subject to ethical and legal scrutiny by ARCs.

### Death is preferable to continued existence in animal facilities

It is often argued that death may be preferable to continued life in research facilities, given the poor living conditions of these animals (e.g., restricted environments, limited enrichment, lack of social bonds). From this perspective, killing is not seen as a harm but as a potential benefit to the animals involved.

However, this line of reasoning diverts attention from the key ethical issue. Rather than justifying killing, such arguments underscore the urgent need to improve animal welfare standards in animal research. It is outside the scope of this section to go into detail regarding the relationship between animals’ living conditions and the validity of animal research.^27^ That being said, if an animal’s life is considered not worth living in standard conditions, it should raise serious concerns about the legitimacy of the research environment itself, rather than legitimizing killing as a solution.

While we acknowledge that rehoming all animals in private homes is likely not feasible (and even illegal in the case of genetically modified animals) we claim that efforts should be directed toward funding housing improvements and creating institutional pathways for continued care whenever possible.

### Prohibiting killing for economic and logistic reasons would undermine scientific progress

One could argue that prohibiting the killing of research animals for economic and logistic reasons would dramatically increase the costs of experiments.^28^ As a result, fewer would take place, thus severely limiting scientific progress and research benefits for society. From this perspective, such killings are considered a regrettable but necessary part of maintaining a viable research system.

This objection rests on two unproven assumptions. First, that avoiding these killings would necessarily lead to a reduction in the number of experiments. Second, that such a reduction would meaningfully harm scientific progress. Historically, revisions in animal welfare regulations, such as improved housing standards or stricter protection for certain species, have often been met with similar concerns (Rodriguez Perez 2023). Yet scientific progress has continued, often accompanied by the development of alternative methods.

In addition, restricting the unnecessary killings of research animals could encourage more responsible breeding practices and reduce waste. If such a shift leads to fewer but more ethically justified experiments, it could enhance public trust and animal research’s legitimacy (MacArthur Clark et al. 2019).

Finally, it is sometimes argued that stricter regulations would simply displace research to countries with lower ethical standards. This argument is frequently invoked to resist progress in animal welfare regulations (Müller 2024). However, it is also based on an unproven assumption. It could also be the case that Switzerland will instead lead by example, encouraging other jurisdictions to adopt higher standards through diplomatic and academic channels (Blattner 2019b).

## Conclusion

In this article, we argued that the harm of killing is systematically overlooked in current animal research Harm-Benefit Analysis (HBA), which undermines the ethical legitimacy of animal research. We used the case of Swiss animal research laws and guidelines, known to be among the strictest worldwide, to illustrate this point. Our analysis is readily applicably to, and all the more valid for laxer jurisdictions.

We demonstrated that the harm of killing is inadequately considered in Swiss laws and guidelines, which not only creates challenges for professionals (e.g., researchers and animal research commissions) having to assess the harms of experiments but also leads to the trivialization of killing in practice and undermines the ethical legitimacy of animal research.

Our analysis reveals that among the four types of harms of killing that should be considered once we recognize the inherent worth and wellbeing of sentient animals, most are insufficiently regulated in Swiss laws and guidelines. We showed that all killings involve a harm to the inherent worth of the animal as a fundamental deprivation. Moreover, we argued that some killings involve additional harm regarding wellbeing (i.e., deaths involving sentientist harms and deprivation of future wellbeing). Both of these types of harms are not explicitly recognized in the Swiss context – or most other legislations, for this matter. The fourth type of harm we defined is mentioned in Swiss guidelines, namely that some killings involve a greater failure to respect the animals’ inherent worth. In particular, we discussed the killing of animals for economic and logistic reasons as a concerning manifestation of this problem, especially genetically modified animals who are bred but not used in research.

This failure to recognize all harms of killing results in its trivialization in practice and undermines the ethical legitimacy of current animal research in the Swiss context and, mutatis mutandis, elsewhere. We illustrated this by presenting two cases (i.e., genetically unfit animals and so-called “leftover animals”) which demonstrate how economically driven killings, though unnecessary and ethically questionable, remain common and legally permitted in animal research. To address this issue, we made the following suggestions. First, killing should always be subject to ethical and legal scrutiny by animal research commissions. Second, killings should pass the necessity test (i.e., killing should be permitted if, and only if, it is necessary to achieve the scientific purpose of the experiment). Third, (all) killing(s) should be considered in HBAs.

Although our focus has been on Switzerland, our analysis is relevant to any country using the HBA to evaluate animal research projects. Whether or not a jurisdiction formally distinguishes between sentientist and non-sentientist harms, neglecting the harm of killing undermines the legitimacy of HBAs and, ultimately, animal research. Jurisdictions with similarly detailed legislation, as well as those with less developed frameworks, could benefit from implementing the recommendations proposed here. In doing so, this article contributes to ongoing international efforts to professionalize the field of animal research ethics, in parallel with similar developments in human research ethics.

## Data Availability

Not applicable.

## Footnotes

1 We will use “animal research” and “animal experiment” interchangeably.

2 Other examples of countries with the notion of animal dignity in their legislation are the Liechtenstein Animal Welfare Act (Liechtensteinische Landesverwaltung 2010): ‘The purpose of this Act is to protect the dignity and welfare of animals’ (Art.1).; Article 1 of the Luxembourg Animal Protection Act of 27 June 2018 (Grand Duchy of Luxembourg 2018): ‘The purpose of this Act is to ensure the dignity, protection of life, safety and welfare of animals.’; Art. 47(1) of the South Korean Animal Protection Act (Republic of Korea 2023): ‘Animal testing shall be conducted in consideration of the enhancement of human welfare and the dignity of animal life.’

3 Under Swiss law, *wellbeing* refers to a state in which animals are clinically healthy, free from pain, suffering, and anxiety, and able to express species-specific behaviors. We use wellbeing in this sense, without distinguishing it from welfare.

4 We will use the term “euthanasia” when death is in the animal’s best interest, e.g., due to unbearable suffering at the end of life. We use the term “kill” to describe deaths that are not in the animal’s best interest but are carried out for other purposes, such as tissue collection or economic reasons.

5 Further frameworks include Nussbaum’s capabilities account, which establishes her ten capabilities as the minimum standard to be met in animal research until the ‘moral tragedy’ of animal research is brought to an end by non-animal alternatives (Nussbaum 2023); and Crary and Gruen’s critical ecofeminist account, which emphasises the structural injustices that occur in animal research (Crary and Gruen 2022). We thank an anonymous reviewer for highlighting these texts.

6 See footnote 2.

7 Cst. Art. 120, para. 2.

8 The European Union uses the following notion in article 12 of 2010/63/EU: “Animals have an intrinsic value which must be respected.” For argumentative purposes, we assume that inherent worth in the Swiss legislation is equivalent to intrinsic value in the EU Directive.

9 AniWA art. 2, para. 1.

10 Note that we do not consider the death of animals whose lives are overall not worth living (and may require euthanasia) as a harm to their wellbeing.

11 Legal scholarship discussing Articles 80 and 120 para. 2 of the Swiss Federal Constitution highlights that the constitutional protection of animals extends beyond the prevention of sentientist harms, to include respect for the intrinsic value and dignity of living beings. This has led commentators to argue that the killing of an animal, even without causing pain, may constitute a legally relevant harm or violation of dignity (Erras 2023; Erras and Rainer 2023). Here we will focus on how these constitutional values are implemented in animal research law and practice, with particular attention to the HBA.

12 We use the English version provided by the Swiss government. Note that only the German, French, and Italian versions of the law are legally binding.

13 In Swiss guidelines, it is rather the “(non)-pathocentric” terminology that is used. Here we use “(non-)sentientist” interchangeably with “(non-)pathocentric”.

14 AniWA, Art. 3 lit. a.

15 AniPO, Art. 136 para. 2.

16 Some examples of sample collection and surgical interventions that correspond to each severity degree: No harm: Single collection of blood sample; Mild harm: Skin biopsies; Moderate harm: Gavage; Severe harm: Implantation of catheters in the abdominal aorta.

17 Here, we use the term “harm”, while the non-binding English version of Swiss laws use “strain” or “constraint” in different context. The German, French, and Italian versions of the law are legally binding, but they show inconsistencies (e.g., AEO, Art. 24: the French term “Contrainte” and the German equivalent “Belastung” are inconsistent with the Italian term “Sofferenza” which indicates suffering rather than mere constraint). For simplicity, we will continue using the term “harm” to refer these different aspects.

18 Euthanasia in this context does not necessarily fit our definition, as it is not clarified that the killing is in the animal’s best interests.

19 AniWA, Art. 21 outlines the legal requirements for the killing (slaughter) of animals.

20 AniWA, Art. 17. AniWA Art. 18 para. 1 and para. 3.

21 Here, we use the term “involving harm” in reference to AniWA Art. 17, which describes such procedures as those that “inflict pain, suffering or harm,” “induce anxiety,” “substantially impair general well-being,” or “may disregard the animals’ dignity” (quoted from the non-binding English version).

22 AniPO, Art. 139 para. 4, *e contrario*.

23 AniWA, Art. 34.

24 This problem (i.e., categorizing cases of excessive instrumentalization as severity degree 0) alone would require an extensive analysis, as it also suppose a violation of the AniWA, but it is not the purpose of this paper to do it.

25 AniWA art. 19, para 4, art. 17.

26 Nonetheless, we acknowledge that more fundamental critiques question the reliance on HBAs and, more broadly, the ethical legitimacy of animal research. As noted above, alternative ethical frameworks have been proposed to move beyond harm-benefit quantification and sentience-based considerations. These perspectives could give rise to additional objections not addressed here, including the view that the killing of animals in research cannot be justified. Engaging fully with these broader critiques is beyond the scope of this paper, though. Instead, we remain within existing governance frameworks and examine how the harm of death can be more adequately recognized and incorporated within them. We thank an anonymous reviewer for drawing our attention to this point.

27 In recent decades, studies have reported the link between animal welfare and scientific validity, emphasizing the need for better animal welfare in research for example through housing enrichment. See for example Bayne and Würbel (2014).

28 Rehoming animals is resource intensive for facilities, time consuming for staff, and adoption options are limited (Skidmore and Roe 2020). Creating more options, or simply caring for the animals in the facility, could increase costs not only in terms of resources, but also of staff salary.
